# Narrowing Down the Mapping of Plant Sex-Determination Regions Using New Y-Chromosome-Specific Markers and Heavy-Ion Beam Irradiation-Induced Y-Deletion Mutants in *Silene latifolia*

**DOI:** 10.1534/g3.111.001420

**Published:** 2012-02-01

**Authors:** Naoko Fujita, Chihiro Torii, Kotaro Ishii, Wataru Aonuma, Yuji Shimizu, Yusuke Kazama, Tomoko Abe, Shigeyuki Kawano

**Affiliations:** * Department of Integrated Biosciences, Graduate School of Frontier Sciences, The University of Tokyo, Chiba, 277-8562 Japan; † RIKEN Nishina Centre, Saitama, 351-0198 Japan

**Keywords:** *Silene latifolia*, XY, sex chromosomes, STS marker, simple sequence repeat

## Abstract

*Silene latifolia* is a well-studied model system for plant XY sex determination. Three maleness factors are thought to function on the Y chromosome, gynoecium suppression factor (GSF), stamen-promoting factor (SPF), and male fertility factor (MFF), and their deletions result in hermaphrodites, anther defects, and pollen defects, respectively. Although a framework map of the Y chromosome exists, the sex determination genes have not been identified, and no markers close enough to potentially be used for BAC library screening are yet available. The analysis of Y deletion mutants by Y-chromosome-specific STS markers is an efficient way to isolate sex determination regions, but more Y-specific STS markers are needed to accelerate the exploration of sex determination factors. Herein, we report a marker design method that uses simple sequence repeats, which is especially effective on the Y chromosome of *S. latifolia* because it contains many simple sequence repeats. Six new Y-chromosome-specific STS markers were obtained, SmicSy1–6. These were used to detect relatively small Y deletion sites in heavy-ion beam irradiation-induced mutants. The mapping of male sex determination regions was narrowed down by using more markers and smaller-sized Y deletion mutants. One new marker, SmicSy6, is a proximal marker to SPF and, thus, a second index for SPF. The region including SPF is thought to be located between two SPF proximal markers. The flower phenotype correlates with the deletion size of SPF using SPF proximal markers. These findings represent new progress in isolating the sex determination factor, which has been studied for more than 50 years.

The sex of the dioecious plant *Silene latifolia* is determined by XY sex chromosomes, and morphological male/female differences are seen in the floral organs. The male flower (XY) has three characteristics controlled by sex-determining factors on the Y chromosome (Westergaard 1958). The gynoecium is suppressed by the gynoecium-suppressing factor (GSF), whereas the flowers of XX females have five styles. The stamen-promoting factor (SPF) leads to development of 10 stamens in the male plant, whereas none are developed in the female plant. Finally, presence of the male fertility factor leads to anther maturation and promotes fertile pollen.

Deletion of any of the sex-determining regions causes abnormal stamen/gynoecium phenotypes. A defect in the GSF leads to a hermaphrodite phenotype (Westergaard 1958). In a normal male flower, the gynoecium is suppressed, but the hermaphrodites develop both gynoecium and stamens. An asexual mutant (early-stage stamen-suppressed mutant [ESS] in this study) results from a defect in the SPF. ESS mutants show neither development of gynoecium nor stamens, whereas GSF functions normally, suppressing the gynoecium. In the stamen defect mutant (intermediate-stage stamen-suppressed mutant [ISS] in this study), because of a partial deletion of the region containing the male fertile factors functioning at intermediate stage, an inhibition of stamen maturation such as filament elongation arrest occurs, which results in the expression of immature stamens. The pollen defect mutant (late-stage stamen-suppressed mutant [LSS] in this study) forms sterile stamens. Pollen development is also affected with an anther dehiscence defect and/or sterile pollen production.

Approximate chromosomal locations of these three sex-determining regions have been inferred by Y-chromosome deletion mutant analysis using sequence tagged site (STS) markers (Bergero *et al.* 2008). MK17 (Hobza *et al.* 2006), a marker that is deleted in most hermaphrodites, is the marker located closest to GSF. ScQ14 (Zhang *et al.* 1998) is inferred to be near SPF because it is deleted in most asexual mutants. Bacterial artificial chromosome (BAC) screens using MK17 or ScQ14 may contain sequences of these sex-determining regions, which could allow the genes involved to be sequenced.

In *S. latifolia*, which has a large Y chromosome (estimated to be 570 Mb in total; Liu *et al.* 2004), Y-chromosome-specific STS markers are key tools in isolating the sex-determining regions. Previously, deletion sites have been detected by this method (Bergero *et al.* 2008; Lebel-Hardenack *et al.* 2002; Zluvova *et al.* 2007) and used to develop a framework of the Y-chromosome map, including locating the sex-determining regions.

To further refine the map of the areas containing the sex-determining regions, a more effective method of identifying Y-chromosome-specific markers is needed. In the current study, we used a method using compound microsatellites (Lian *et al.* 2001, 2006) with a primer design based on two microsatellites arranged in tandem. Fluorescence *in situ* hybridization (FISH) analysis with microsatellites as probes indicated that the *S. latifolia* genome includes a high percentage of microsatellites (Kejnovsky *et al.* 2009; Kubat *et al.* 2008). FISH analysis with mono-, di-, and tri-nucleotide microsatellites as probes detected dispersed microsatellites in the *S. latifolia* genome, especially on the Y chromosome (Kubat *et al.* 2008). A survey of repetitive sequences by screening of *S. latifolia* shotgun genomic library (Cermak *et al.* 2008) estimated that only 1.4% of sequenced clones contained microsatellite loci in the genome as a whole, probably because the microsatellites have only short repeats. However, on the basis of the distribution of microsatellites on the Y chromosome estimated by measuring signals in FISH analysis (Kejnovsky *et al.* 2009), microsatellites can be expected to represent at least 30% to 40% of the Y-chromosome length at metaphase. The *S. latifolia* Y chromosome should therefore contain many such markers. Here, we use tandemly arranged microsatellites to use the switching points between them as unique markers. Y-chromosome-specific sequences were therefore isolated using multiple compound simple sequence repeat (SSR) primers and tested to ascertain whether the fragment could be used as a STS marker, generating six new Y markers.

The new Y-linked markers were tested on 17 Y-chromosome deletion mutants obtained by heavy-ion beam irradiation and γ irradiation. In previous studies, Y-chromosome deletion mutants were induced by X-irradiation (Lebel-Hardenack *et al.* 2002) and γ irradiation (Zluvova *et al.* 2007). An advantage of mutation induction by heavy-ion beam irradiation is the ability to induce small deletion sizes of less than 1 kb (Kazama *et al.* 2007, 2008). This new source, heavy-ion beam irradiation, was suggested to have the potential to create new mutant phenotypes. In this study, we used carbon-ion, iron-ion beam, and γ irradiation to induce mutations. Seventeen Y chromosome deletion mutants were produced by either carbon-ion beam irradiation or γ irradiation. These deletion mutants were then screened for deletion sites using Y-chromosome-specific STS markers as well as our six new Y-linked markers. Our Y-chromosome-specific STS markers indeed allowed us to detect deletions that were not detectable with previously used markers. Therefore, this new approach narrows down the Y regions deleted and can be used for more precise Y-chromosome mapping than has previously been possible.

## Materials and Methods

### Plant materials

An inbred *S. latifolia* K-line (“K” indicates that this inbred line was grown at Kashiwa campus, Tokyo University) of 11 generations of brother/sister mating (Kazama *et al.* 2003) was used. Plants were grown from seeds in pots in a regulated chamber at 23° with a 16-hr light/8-hr dark cycle. The Y deletion mutants were obtained by either heavy-ion beam irradiation or γ irradiation of seeds or pollen, and 1008 of these plants were grown in a field. Each flower’s phenotype was screened, and plants with defects in their gynoecium and/or stamen were isolated and used as Y-chromosome deletion mutants. Genomic DNA was extracted from fresh leaves using a Nucleon Phytopure Genomic DNA Extraction Kit (GE Healthcare, Milwaukee, WI) according to the manufacturer’s instructions.

### DNA library construction

An adaptor-ligated DNA library was constructed as described by Lian *et al.* (2001). Male genomic DNA (4 μg) was digested with 20 U of a restriction enzyme (*Eco*RV, *Dra*I, or *Ssp*I). The restricted fragments were ligated with a specific blunt adaptor (48 mer: 5′-GTAATACGACTCACTATAGGGCACGCGTGGTCGACGGCCCGGGCTGGT-3′ and an 8-mer with the 3′-end capped by an amino residue: 5′-ACCAGCCC-NH_2_-3′) by use of a DNA ligation kit (Takara Bio, Otsu, Japan). DNA libraries were constructed from each of the three different restriction enzymes and stored at –20°.

### Design of specific primers from amplified fragment compound SSR primers

Fragments were amplified from the *Eco*RV, *Dra*I, and *Ssp*I DNA libraries using an adaptor primer AP2 (5′-CTATAGGGCACGCGTGGT-3′; Lian *et al.* 2001, 2006) and 20 types of compound SSR primers consisting four arrangements of the five combinations listed in Table 1. Polymerase chain reaction (PCR) amplification was performed using Blend Taq polymerase (Toyobo, Tokyo, Japan) in a Thermal Cycler Dice TP600 (Takara Bio). The conditions were 5 min at 94°, followed by 25 cycles of 30 s at 94°, 30 s at 60°, and 1 min at 72°, with a final extension of 5 min at 72°. The amplified products were electrophoresed on 1% agarose gels. The remaining PCR products were purified by a Montage PCR Centrifugal Filter Device (Millipore, Billerica, MA) and subcloned using a TOPO TA Cloning Kit (Invitrogen, Carlsbad, CA) according to the manufacturer’s instructions.

**Table 1:** List of compound SSR primers used in this study

	SSR-1	SSR-2	SSR-3	SSR-4	SSR-5
21-mer Simple (21S)	(CAG)_4_(CAA)_3_	(CAG)_4_(CAT)_3_	(CAG)_4_(GAA)_3_	(CAA)_4_(GAA)_3_	(CAA)_4_(TAA)_3_
21-mer Modify (21M)	AG(CAG)_3_(CAA)_3_C	AG(CAG)_3_(CAT)_3_C	AG(CAG)_3_(GAA)_3_G	AA(CAA)_3_(GAA)_3_G	AA(CAA)_3_(TAA)_3_T
27-mer Simple (27S)	(CAG)_5_(CAA)_4_	(CAG)_5_(CAT)_4_	(CAG)_5_(GAA)_4_	(CAA)_5_(GAA)_4_	(CAA)_5_(TAA)_4_
27-mer Modify (27M)	AG(CAG)_6_(CAA)_2_C	AG(CAG)_6_(CAT)_2_C	AG(CAG)_6_(GAA)_2_G	AA(CAA)_6_(GAA)_2_G	AA(CAA)_6_(TAA)_2_T

SSR, simple sequence repeat.

The plasmids were transformed into *Escherichia coli*. Single colonies were used as templates and the cloned fragments were amplified using M13 primers. The inserted fragment lengths were verified by 1.5% agarose gel electrophoresis. Amplified fragments were purified using Millipore Multi Screen Vacuum Manifolds (Millipore) and MultiScreen HTS plates (Millipore), and sequenced using an ABI PRISM3130x Genetic Analyzer (Applied Biosystems, Foster City, CA). For each fragment containing the compound SSR sequences and the adaptor sequences, specific primers were designed using Primer 3 software.

### Isolation of the Y-chromosome-specific STS markers (SmicSy)

In total, 578 specific primers were tested for male specificity. PCR was performed using a specific primer with an appropriate compound SSR primer. Electrophoresis patterns between male and female genomic templates were then compared. The primer sets amplifying single or double bands were named ‘SmicS’ primers (STS marker in combination with SSR primer). SmicS primers yielding a male-specific band were inferred to amplify Y-chromosome-specific regions, and the bands were isolated; these were named SmicSy. The optimum annealing temperature for each SmicSy was determined by gradient PCR using annealing temperatures from 50° to 65°. The compound SSR specificity was also checked with arrangement patterns other than 27M, *i.e.*, 21S, 21M, and 27S arrangement patterns. The thermocycling profile was 5 min at 94°, followed by 25 cycles of 30 s at 94°, 30 s at the appropriate annealing temperature, and 1 min at 72°, with a final extension for 5 min at 72°.

### Y-chromosome deletion mapping

In total, 19 Y deletion mutants were used for Y-chromosome deletion mapping in this study: 17 newly induced mutants in addition to K034 (Koizumi *et al.* 2007) and R025 (Koizumi *et al.* 2010). Each 25 µl reaction mixture contained 50 ng of template DNA, 2.5 µl of 10× dNTPs, 10× Blend Taq Buffer, 0.2 µl of Blend Taq, and 1 µl of each SmicSy primer at 10 µM. A specific primer was used as a forward primer, and compound SSR primer was used as a reverse primer. PCR cycling was the same as described previously. The reaction products (8 μl) were electrophoresed on 1.5% agarose gels. After staining with ethidium bromide, fragments were visualized on a UV illuminator (Atto, Tokyo, Japan). Wild-type (WT) male and female genomic DNA was used as controls. PCR detection was repeated three times for each STS marker.

## Results

### Design of Y-chromosome-specific markers with compound SSR

**Figure 1:**
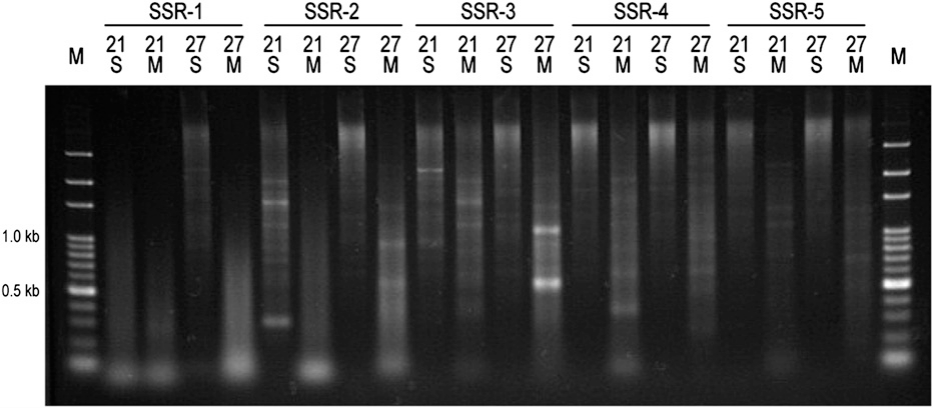
Amplified fragments from an adaptor-ligated male genomic DNA library. PCR amplification was performed with compound SSR primer as the forward primer and an adaptor primer as the reverse primer. Twenty compound SSRs consisting of five different microsatellite combinations (SSR-1 to -5) and four different arrangement patterns (21S, 21M, 27S, and 27M) for each SSR combination are indicated at the top. The electrophoretic patterns differ among the four arrangement patterns within the same SSR combination. M indicates DNA size marker.

The three base microsatellites chosen for this study were determined following the work of Kejnovsky *et al.* (2009). Five different microsatellite combinations that are highly conserved on the Y chromosome were chosen. The compound SSR primers in this study is consisted of five different combinations of two tandemly arrayed microsatellites: (CAG) and (CAA), (CAG) and (CAT), (CAG) and (GAA), (CAA) and (GAA), and (CAA) and (TAA). (CAG) is chosen for the first three combinations because strong signals on the Y chromosome were detected in FISH analysis with (CAG)_10_ as a probe (Kubat *et al.* 2008). (CAA) for the last two combinations was chosen for the same reason. For each combination, two different repeat lengths (21 and 27 mer) were chosen. In addition, two structures were tested for each 21 and 27 mer: a “simple” type of arrangement, which simply repeated the three unit bases [21 mer Simple: 21S, 27 mer Simple: 27S; *e.g.*, SSR-1_21S: (CAG)_4_(CAA)_3_], and a modified type, to which partial microsatellite units were added at the 3′ and 5′ ends [21 mer Modify: 21M, 27 mer Modify: 27M; *e.g.* SSR-1_21M: AG(CAG)_3_(CAA)_3_C; Table 1]. Despite the microsatellite units were the same (21S, 21M, 27S, 27M), the fragments amplified with primers for the four arrangements differ, in addition to different bands being produced by the different microsatellite combinations (Figure 1).

**Figure 2:**
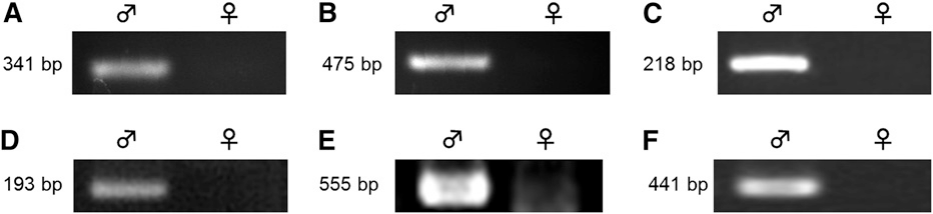
Male specificity of new Y-chromosome-specific markers, SmicSy1–6. SmicSy1 (A), SmicSy2 (B), SmicSy3 (C), SmicSy4 (D), SmicSy5 (E), and SmicSy6 (F). The fragment was only amplified with the male genomic DNA template (♂); no fragment was amplified with the female genome (♀). Fragment sizes are indicated on the left.

In total, 578 specific primers were designed from within the flanking sequences obtained. Each specific maker with appropriate compound SSR primers was examined the male specificity. If a fragment is amplified both in male and female, that marker should be located on autosome, X chromosome, or pseudo-autosomal region of XY chromosomes. A marker specific for male should be located on the Y chromosome. Markers amplifying single or double bands in both male and female were named SmicS (STS marker in combination with SSR primer). Forty SmicS were used to examine whether non-Y-linked regions were deleted in obtained mutants; as a result, several deletions were found using SmicS (data not shown). This finding suggests that Y deletion mutants are deleted in not only the sex determination region on Y but also regions on autosomes or X. Male specific primer sequences are listed in Table 2. SmicSy1 and SmicSy2 were designed with longer sequences to increase their Y-chromosome specificity. The annealing temperatures determined for each primer by gradient PCR are given in Figure 2. In total, six male specific markers were obtained. The overall proportion of markers per primer set was thus only 1%. However, with the compound SSR primers such as SSR4_27M and SSR5_27M, the yield was much greater (6%–10%). SSR4_27M and SSR5_27M, both including (CAA), were highly efficient in obtaining Y-chromosome-specific markers. Despite the compound SSR primers targeting the switch points between different microsatellites, most of the sequences amplified were not microsatellites, suggesting that few tandemly arrayed microsatellites exist in the genome of *S. latifolia*.

**Table 2:** Primer sequences for SmicSy1-6

SmicS	Specific Primer*^a^*	SSR Primer*^b^*	Ta (°C)
SmicSy1	CTCACCGTAGCCGAGAAGAAGGAGAAAGG	SSR-5_27M	60
SmicSy2	TGTCGATCGTTCAAAGCAACTACAGG	SSR-5_27M	60
SmicSy3	GCTCCCAACACTACGCCTTA	SSR-5_27M	63
SmicSy4	GCAAATGAAATCATCTCGACTG	SSR-4_27M	63
SmicSy5	AGTCGAGAGGCACGAAAATG	SSR-4_27M	58
SmicSy6	CCATTTCAATTTGGGGTTTG	SSR-4_27M	60

SSR, simple sequence repeat; Ta, annealing temperature.

a Specific primer as a forward primer.

b SSR primer as a reverse primer.

### New Y chromosome-specific markers (SmicSy)

The male specificity of these markers was then examined. Six male-specific SmicS (Figure 2) were isolated and named SmicSy (STS marker in combination with SSR primer y chromosome-specific). One possible reason that the Y markers were easy to find is that the Y chromosome contains numerous (CAA) simple repeat sequences. It can be inferred from the FISH analysis with (CAA)_10_ as a probe (Kubat *et al.* 2008) found strong signals on the Y chromosome, while only weak signals were detected on the X chromosome and autosomes.

We attempted to design SmicSy1–6 markers with both primers specific instead of using compound SSR primer. However, across a wide range of annealing temperatures, the male specificity of our markers was more stable in combination with a compound SSR primer for one side, than with specific primers for both sides. The 21S, 21M, 27S SSR primers yielded only nonmale-specific markers. Of the compound SSR primers tested, the only ones that led to male-specific SmicSy markers were SSR-4_27M and SSR-5_27M.

**Figure 3:**
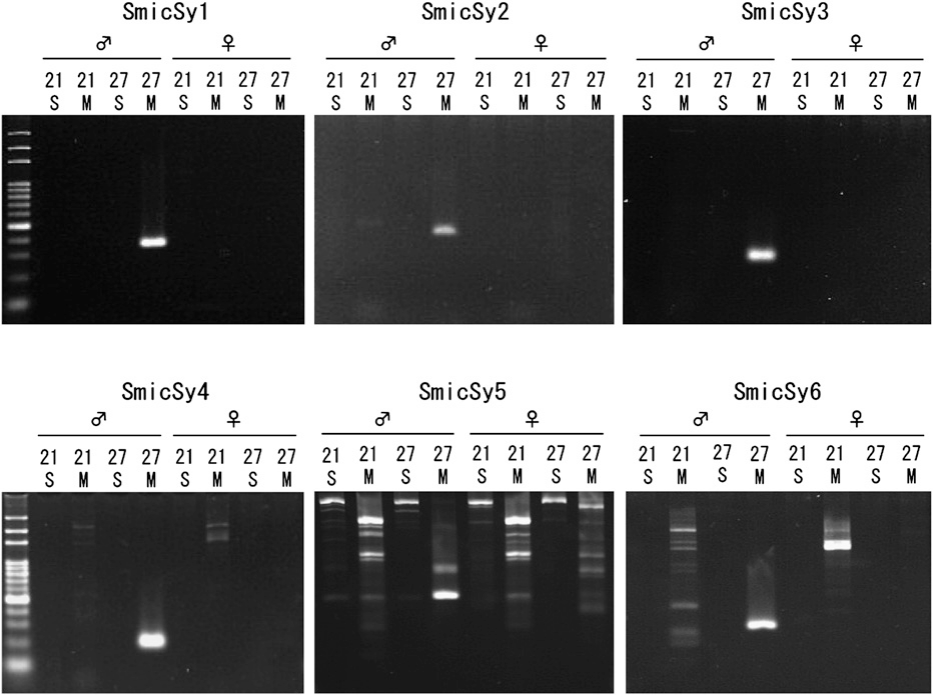
SmicSy PCR amplification in combination with appropriate compound SSR primers in the arrangement pattern. Male-specific fragments were only amplified with the appropriate 27M compound SSR primer. No fragment was amplified from the female genome in SmicSy 1–3, on the top. Nonspecific fragments were amplified with different arrangement patterns of compound SSR primers, but the male-specific fragment was only amplified with the 27M arrangement pattern (SmicSy 4–6). DNA size marker is shown on the left.

Two primers, SSR-4 and SSR-5, yielded all six SmicSy1–6 markers and were more efficient in producing Y markers than the other 18 primers. The six SmicSy markers involving SSR-4 and SSR-5 were tested to determine whether the same fragment amplified with different arrangements of the same compound SSR primers (*i.e.*, 21S, 21M, and 27S; Figure 3). The SmicSy1–3 fragments were amplified using other arrangements, but the male-specific fragment was again only obtained with the 27M pattern.

### Isolation of asexual and hermaphrodite mutants induced by heavy-ion beam irradiation and γ irradiation

**Figure 4:**
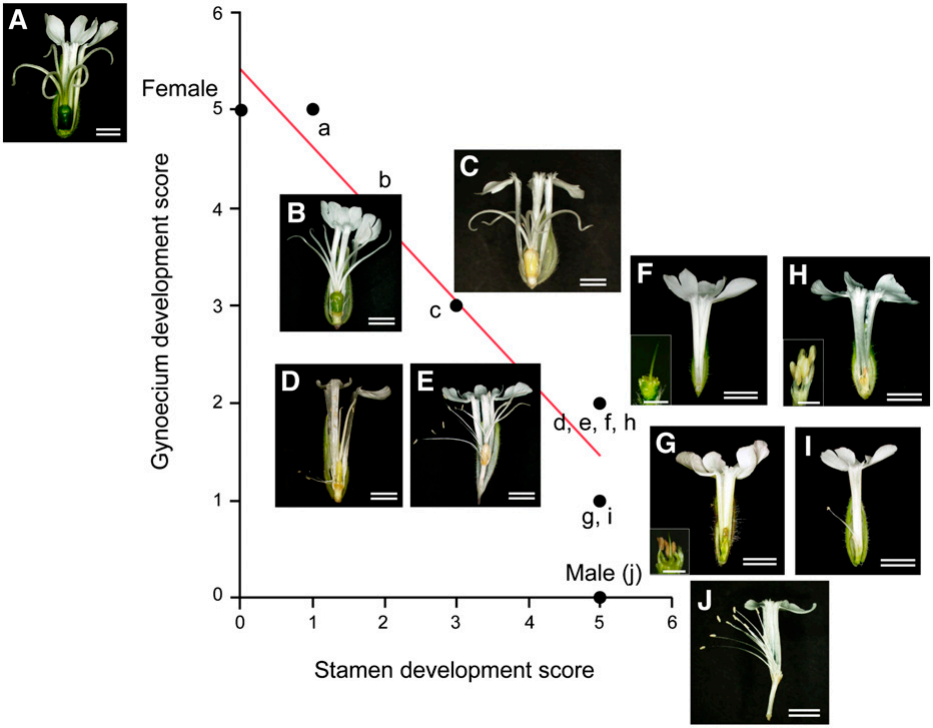
Correlation between the gynoecium and stamen developments. WT female flower (A,a), Gynoecium Promoted mutant (hermaphrodite mutant), GP1–4 (B–E, b–e), Early-stage Stamen Suppressed mutant, ESS1 (F,f), Intermediate-stage Stamen Suppressed mutant, ISS (G-I, g–i), and WT male flower (J,j). Note that GP mutants were subdivided into two classes: incomplete hermaphrodite GP1–3 (B-D, b–d) and complete hermaphrodite GP4 (E,e). The inverse relationship between gynoecium and stamen development was seen in the incomplete hermaphrodites (b-d). A mature gynoecium developed in the WT female (a); the gynoecium development level decreased from GP1 (b) to GP2 (c) to GP3 (d). Bar: 3 mm; double bar: 1 cm.

We obtained 17 new mutants by heavy-ion beam irradiation and γ irradiation. These all 17 mutants carry a Y chromosome because at least one of Y-linked marker is present. These mutant phenotypes were classified into two groups: a hermaphrodite group (Figure 4b–e) and an asexual group (Figure 4f–i). The hermaphrodite group (denoted by “GP”) was further subdivided into “complete” and “incomplete” classes. The asexual mutant group was further subdivided into a complete asexual mutant class (ESS; *e.g.*, Figure 4f) in which stamens did not develop, an incomplete asexual mutant class (ISS; *e.g.*, Figure 4g–i), which have immature stamens, and a fertility defect mutant class (LSS), which have morphologically mature but sterile stamens.

The complete hermaphrodite class had both complete stamen and gynoecium, whereas the incomplete hermaphrodite class had a gynoecium that was at least partially developed. GP1-3, GP7, and GP9 were incomplete hermaphrodites (GP1–3 are shown in Figure 4b–d, respectively). GP1, GP3, and GP9 were obtained by heavy-ion beam irradiation, whereas GP2 and GP7 were obtained by γ irradiation. GP7 and GP9 had unusual phenotypes: GP7 had two styles and 10 stamens, whereas GP9 had five styles on a tiny ovary (unobservable by the naked eye; W. Aonuma, unpublished data). Further investigation of GP7 and GP9 is needed because these mutants were found in the field. These unusual phenotypes may be attributable to environmental or other factors. GP1–3 were found 1 year before GP7 and 9, and grown in the plant cultivation room under stable conditions (23°, 16-hr light/8-hr dark cycle). The flower phenotypes have been observed continuously for 1 year.

Among the incomplete hermaphrodites GP1–3, the filament length and anther maturity of stamens increase and the gynoecium maturity tends to decrease. WT females have a large, green ovary and five thick styles (Figure 4a), whereas a complete hermaphrodite has a smaller pale yellow ovary and thinner styles (GP4, Figure 4e). Changes in ovary colors and size of styles can be seen in three incomplete hermaphrodites (GP1–3, Figure 4b–d). GP1 and GP2 produced no pollen grains. GP1 (Figure 4b) resembles a female flower, having a greenish normal sized ovary and thick styles. GP3 is morphologically similar to a complete hermaphrodite and produced trinuclear pollen grains but self-fertilization failed (data not shown). GP4 is capable of self-fertilization. In the incomplete hermaphrodites (GP1–3), the stamen development increased (*e.g.*, filament lengths, GP1: 4–6 mm, GP2: 10–17 mm, and GP3: 8–28 mm) as gynoecium maturity decreased (GP1 > GP2 > GP3, for details see supporting information, Table S1). This inverse relationship between the gynoecium and stamens was seen in all flowers of each strain observed for 1 year. The phenotypes for each GP mutant are listed in Table S1.

The asexual mutant group was subdivided into three classes (ESS, ISS, and LSS) according to stamen developmental level (Figure 4) and flower developmental stage (Grant *et al.* 1994). A list of asexual mutants is presented in Table S2. In the ESS class, the stamen development stopped at stage 5 or 6, resulting in no morphological sex differences in the primordial flower (Figure 4f). The ISS class developed immature stamens that went to stage 7 or 8 (Figure 4g–i). The LSS class formed morphologically normal stamens, but a fertility defect occurred at stage 9–12, *i.e.*, the pollen development stages. Previously reported pollen fertility mutants (*e.g.*, pf1-5 mutants in Zluvova *et al.* 2007) could be included in this class. We obtained only one LSS mutant (LSS1) in the present study. LSS1 had well-developed stamens, but anther dehiscence was defective; it produced trinucleate pollen grains, but their germination rate was low (12.02%). We have obtained other LSS mutants induced by heavy-ion beam irradiation and γ irradiation; the details of these mutants are currently under investigation in our laboratory.

### Y-chromosome deletions in asexual and hermaphrodite mutants

**Figure 5:**
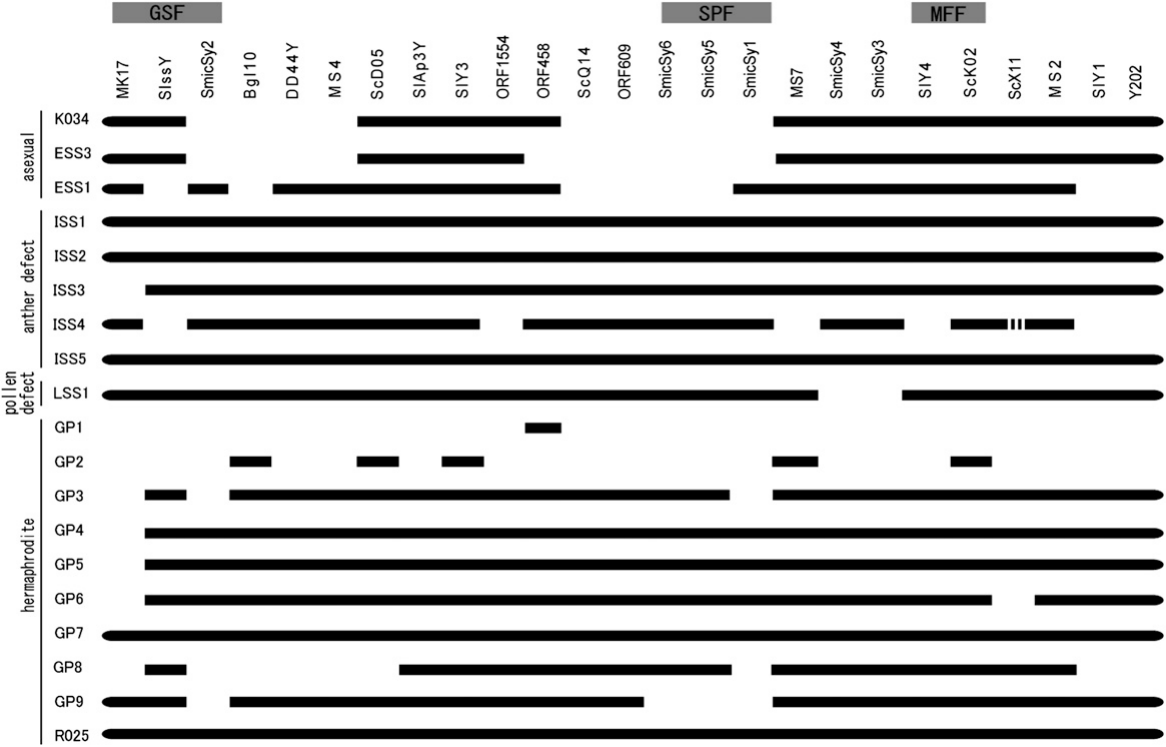
Outline of Y-chromosome deletion mapping. The markers are shown at the top, ordered according to the Y map (Zluvova *et al.* 2007) with new markers SmicSy 1–6 and open reading frame markers obtained from BAC sequences (Y. Kazama, unpublished data). Mapping of the new STS markers was determined to minimize deletion sites. The mutant names and phenotypes are indicated on the left. Full lines denote markers present in each deletion mutant; dashed lines indicate that the amplified fragment was of smaller size than in the control male template. The deletions are indicated by a blank without scale.

The 17 new mutants, as well as mutant R025, were induced by heavy-ion beam irradiation or γ irradiation, whereas K034 was a spontaneous mutant. The Y chromosome deletion regions were determined using previously published Y-chromosome STS markers, plus our new SmicSy1‒6 markers (Figure 5). No deletions were found in five mutants with any of these markers (ISS1, ISS2, ISS5, GP7, and R025). This finding is surprising for R025, which is a complete fertile hermaphrodite mutant (Koizumi *et al.* 2010) and was expected to have a deletion of GSF.

For two mutants, LSS1 and GP9, although no deletions were found using the previously known markers, Y-chromosome deletions were detected using the new markers. LSS1 is a mutant with failure of anther dehiscence and a low rate of pollen germination, suggesting a deletion of male fertility factor. LSS1 shows deletion of two Y-linked markers, SmicSy3 and 4. Therefore, SmicSy3 and 4 are needed to test for other LSS mutants after obtaining new LSS mutants. GP9 has an unusual phenotype (five styles on a tiny ovary), and four markers, SmicSy1, 2, 5, and 6 are deleted.

We mapped the SmicSy markers by minimizing the number of deletions on previously reported Y-chromosome deletion maps (Bergero *et al.* 2008; Zluvova *et al.* 2007). Most of the ESS mutants had deletions of SmicSy1, SmicSy5, and SmicSy6, suggesting that these markers are close to the SPF. This finding is supported their deletion patterns, which are similar to those for ScQ14, the marker previously closest to SPF. However, BACs screened using ScQ14 contain neither SmicSy5 nor SmicSy6. The deletion patterns for SmicSy5 and SmcSy6 are the same in our mutants, but the presence of SmicSy5 depends on *S. latifolia* individual; it is absent in the genome of plant H2005-1-9 (Bergero *et al.* 2007). We therefore mapped SmicSy6 closer to ScQ14 than SmicSy5 because the region closer to SPF should conserved in all *S. latifolia* males. SmicSy5 is deleted in one of strain H2005-1-9, suggesting that the region detecting by SmicSy5 may be dispensable in males.

## Discussion

### Design of Y-chromosome-specific markers With compound SSR

Not only does high homology between some regions of the X and Y chromosomes make Y markers difficult to isolate, repetitive sequences also adds to the difficulty of obtaining and mapping Y-linked markers. For example, FISH analysis with a probe for microsatellites with three-base units yields many signals, suggesting that the microsatellites are interspersed in both sex chromosomes (Kubat *et al.* 2008). However, some specific microsatellite types accumulate more in the Y than the X chromosome. Specifically, (CAA) accumulation is seen only in one region of the X q-arm, and not in the p-arm, whereas it is conserved in the q-arm of the Y chromosome and is also dispersed throughout the entire chromosome. Microsatellites with repeats of (GAA), (TAA), and (TAC) have also accumulated more in the Y than in the X chromosome (Kejnovsky *et al.* 2009).

SSR-1 is a primer designed on the basis of microsatellites (CAA) and (CAG). Moreover, the Y chromosome contains many (CAA) combinations, although Y-specific markers were not found using SSR-1. This may have been attributable to there being an approximately equal density of (CAG) in the Y and X chromosomes. Using SSR-1, -2, and -3 microsatellites, which all contain (CAG), we found no Y chromosome-specific markers. Previous FISH analysis with (CAG)_10_ as a probe showed similar signal levels on the Y, X chromosomes, and autosomes, unlike the analysis performed with (CAA) (Kubat *et al.* 2008). Therefore, compound SSR primers containing (CAG) have a poor chance of amplifying a sequence of the Y chromosome and thus show a low efficiency in marker discovery.

Despite the compound SSR primers targeting the switching point of two different microsatellites, most of the amplified sequences were not microsatellites, suggesting that amplified sequences are not the switching points of tandemly arrayed microsatellites. The compound SSR primers probably amplified the region of short repeats of microsatellites next to another short repeats. If the Y does indeed contain large regions free from repetitive sequences, the new Y chromosome-specific markers may therefore be useful for BAC screening, which may lead to the isolation of the male sex determination regions.

### Stamen defective mutants induced by heavy-ion beam Irradiation and γ irradiation

Within three classes of male-defective mutants (ESS, ISS, and LSS; see *Introduction*), the suppression levels of the stamen defect mutants (ISS) differ in our study, as in previous mutants (Zluvova *et al.* 2007). However, the Y chromosomal deletion pattern differs between the mutants induced by X-irradiation (Lebel-Hardenack *et al.* 2002) and γ irradiation (Zluvova *et al.* 2007) and those induced by heavy-ion beam irradiation or γ irradiation (dosages of 20, 40, and 80 Gy; the radiation conditions for each mutant are shown in Table S1 and Table S2). A notable difference is that in three mutants with stamen defects (ISS1, ISS2, and ISS5; Figure 5), no Y-chromosome deletions were detected using any of the Y-linked markers, whereas at least one marker was deleted in previously reported strains with flower mutations (summarized in Bergero *et al.* 2008). We subdivided stamen defect mutants into ESS and ISS in this study because not only stamen developmental structure differs between ESS and ISS but also Y-deletion patterns in ISS mutants are different from ESS. All three ESS mutants deleted with Y-linked markers inferred to be located near the SPF, whereas three of five ISS mutants had no Y-chromosome deletions were found using the Y-linked markers (Figure 5). ScQ14 is a marker located near the SPF and deleted in all ESS mutants generated by either γ irradiation or heavy-ion beam irradiation. ScQ14 is also deleted in ISS mutants induced by γ irradiation (Zluvova *et al.* 2007), but not in ISS mutants in this study. This may have been attributable to differences in dosages of γ irradiation or sources of mutations, *i.e.*, heavy-ion beam irradiation. The presence of ScQ14 in ISS mutants in this study suggests that ScQ14 is probably located outside the SPF. More than two genes could be acting during stamen development (Bergero *et al.* 2008; Westergaard 1958; Zluvova *et al.* 2007). SPF (called M1 in Bergero *et al.* 2008) promotes early stamen development, and a second male fertility factor M2 may function later. M2 was mapped next to M1/SPF (Bergero *et al.* 2008). ScQ14 was present in ISS mutants probably because these mutants were deleted only in M2. The stamens of ISS mutants initiate and complete the early stages of development, suggesting that SPF is present. Deletion of M2 probably results in arrest of stamen development region at the intermediate stage. This needs to be confirmed by measuring the Y-deletion sizes in *S. latifolia*. Another possible reason that three ISS mutants with defective stamen development (ISS1, ISS2, and ISS5 in Figure 5) had no detectable deletions of any Y-linked markers may be that the male fertility at the intermediate stages is controlled by genes that are not Y-linked (genes affecting stamen development can, of course, also be located on the autosomes and/or X chromosome). SmicS markers for non-Y-chromosome regions could be used to detect such deletions in the future.

### Hermaphrodite mutants induced by heavy-ion beam irradiation and γ irradiation

Almost all hermaphrodites are deleted in MK17 (the exceptions are GP7, GP9, and R025; Koizumi *et al.* 2010). The three exceptional hermaphrodites are incomplete hermaphrodites with deformed gynoecia (normal ovary with two styles in GP7; tiny ovary with 5 styles in GP9), whereas R025 (Koizumi *et al.* 2010) is a complete, self-fertile hermaphrodite with the ability to self-fertilize (Koizumi *et al.* 2010). GSF must therefore be deleted in R025. This deletion does not include MK17 or any other markers, and could be confined to the GSF. GP7 and GP9 are probably deleted not only for GSF, but also other gynoecium development factors. Such factors must exist on the autosomes or the X chromosome since the gynoecium is developed in the WT female (XX). Deletion of any of factors affecting the gynoecium development may result in a deformed gynoecium, with unusual style numbers and/or failure of ovary development. R025 was shown to have a Y chromosome mutation by crossing (Koizumi *et al.* 2010), whereas the presence of Y chromosome mutations in GP7 and GP9 are unknown because of the failure of self-fertilization. GP7 and GP9 could be caused by autosomal mutation or deletions of other genes in the network interacting with GSF.

### Degree of stamen/gynoecium development

In the other incomplete hermaphrodites and ISS mutants, the development of the stamen and gynoecium are highly variable. Interestingly, hermaphrodite mutants with better-developed gynoecia tend to develop less-mature stamens, and *vice versa*, mutants with plants whose stamens are nearly normal tend to have only a rudimentary gynoecium. GP1 and GP2 (with good gynoecium development and poor stamen development) have deletion of many Y-linked markers (Figure 4, b and c and Figure 5). Although the physical sizes of the Y deletions are not known (but only the numbers of markers deleted), GP1 probably has a large deletion because 24 of the 25 Y markers tested are deleted, and similarly GP2 has 20 markers deleted (Figure 5). Inverse relationships were seen between gynoecium and stamen development in GP1, GP2, and GP3 (Figure 4, b–d). It is clear from correlation between the gynoecium and stamen development scores (Figure 4, for details of each mutant phenotype see Table S1). Although the ovaries of GP1 and GP2 were almost the same size as those of WT females (Table S1), the color differed between GP1, GP2, and WT females (yellowish green in GP1, yellow in GP2, and green in the WT female). The ovary color sometimes changed from green to yellow in WT females, probably because of the nutrient state or other environmental factors. However, the ovaries of GP1–3 did not change to healthy green. The ovary size was indeed smaller in GP3 (WT female: φ4–6 mm; GP1: φ4–5mm, GP2: φ4–5mm, and GP3: φ;2–3mm) and the stamen length was longer (WT female: 0 mm; GP1: 4–6 mm; GP2: 10–17 mm; and GP3: 8–28 mm). In addition, the length of the gynophore (stalks bearing the gynoecium and stamens above the receptacle), which is a male phenotype, increased in the order WT female < GP1 < GP2 < GP3 (Table S1).

The question remains regarding R025, which has complete female as well as male function and no Y-linked marker deletions have yet been found, which does not seem to support the inverse relationship between gynoecium and stamen development. Although R025 has full female and male function, the size of the gynoecium primordia is intermediate between those in females and males (K. Yamanaka, unpublished data). The sizes of the floral meristem (diameter inside the sepal) at stage 4 were not significantly different among WT females, WT males, and R025 (WT female: 137.74 ± 18.77 µm, WT male: 131.13 ± 19.18 µm, and R025: 115.27 ± 8.28 µm, ± SD, *t*-test, *P* < 0.05), suggesting the same floral meristem size for WT females, WT males, and R025. The sizes of whorle 4 (the gynoecium primordia), however, significantly differed at stage 7 (WT female: 309.78 ± 33.14 µm, WT male: 45.49 ± 7.34 µm, and R025: 236.95 ± 45.67 µm, ± SD, *t*-test, *P* < 0.05), suggesting that the primordial gynoecium size in R025 is intermediate between those in WT females and WT males. This inverse relationship was also reported previously in the cryptic dioecious plant, *Thalictrum pubescens* (Davis 2002). As resource investment in one sexual function increases, investment in the other sexual function would necessarily decrease, generating trade-offs among sexual traits. The primordial gynoecium size differences may be an example of trade-offs in *S. latifolia*. The inverse relationship between stamen and gynoecium development suggests the interesting conclusion that the Y-chromosome probably carries many factors affecting flower sex structures, and that at least some of the factors promoting stamen development must suppress the gynoecium, suggesting the predicted trade-offs.

## Supplementary Material

Supporting Information
