# Supplementary material for: Narrowing Down the Mapping of Plant Sex-Determination Regions Using New Y-Chromosome-Specific Markers and Heavy-Ion Beam Irradiation-Induced Y-Deletion Mutants in Silene latifolia
Source: G3 (Bethesda). 2012 Feb 1;2(2):271–8. doi: 10.1534/g3.111.001420 (PMC3284334; doi:10.1534/g3.111.001420)
Supplement: Supporting Information [file supp_2_2_271__index.html]

Supporting Information 

# Narrowing Down the Mapping of Plant Sex-Determination Regions Using New Y-Chromosome-Specific Markers and Heavy-Ion Beam Irradiation-Induced Y-Deletion Mutants in *Silene latifolia*

## Supporting Information for Fujita *et al.*, 2012

**Files in this Data Supplement:**

- Supporting Information - Table S1 and S2 (PDF, 61 KB)
- Table S1 - List of Hermaphrodite mutants (PDF, 61 KB)
- Table S2 - List of asexual mutants in this study (PDF, 52 KB)
