# Supplementary material for: Narrowing Down the Mapping of Plant Sex-Determination Regions Using New Y-Chromosome-Specific Markers and Heavy-Ion Beam Irradiation-Induced Y-Deletion Mutants in Silene latifolia
Source: G3 (Bethesda). 2012 Feb 1;2(2):271–8. doi: 10.1534/g3.111.001420 (PMC3284334; doi:10.1534/g3.111.001420)
Supplement: Supporting Information [file supp_2.2.271_TableS1.pdf]

Table S1 List of Hermaphrodite mutants

Genotype	Mutagen source	Male phenotypes: Stamen number	Filament length (mm)	Gynophore length (mm)	Stamen development score	Female phenotypes: Style number	Style length (mm)	Ovary size (diameter, length in mm)	Ovary color	Gynoecium development score
**Wild-type male**	**n/a**	**10 mature**	**17~33**	**10-14**	**5**	**0**	**0**	**0**	**n/a**	**0**
**GP9***	**C-ion 20 Gy**	**10 mature**	**17~33**	**8~10**	**5**	**5**	**16~20**	**0?**	**n/a**	**1**
**GP8**	**γ 40 Gy**	**10 mature**	**25~32**	**7~8**	**5**	**5**	**20~23**	**2~3, 5~7**	**Green**	**2**
**GP7***	**γ 40 Gy**	**10mature**	**17~33**	**8~10**	**5**	**2**	**16~20**	**0?**	**n/a**	**1**
**GP6†**	**C-ion 20 Gy**	**10 mature**	**25~32**	**7~8**	**5**	**5**	**20~23**	**2~3, 5~7**	**Green**	**2**
**GP5†**	**γ 20 Gy**	**10 mature**	**25~32**	**3~4**	**5**	**5**	**20~23**	**2~3, 5~7**	**Green**	**2**
**GP4†**	**C-ion 20 Gy**	**10 mature**	**25~32**	**7~8**	**5**	**5**	**20~23**	**2~3, 5~7**	**Yellow**	**2**
**GP3***	**C-ion 20 Gy**	**[illegible]10 immature**	**8~28**	**3~4**	**3**	**5**	**20~24**	**2~3, 6~8**	**Yellow**	**3**
**GP2***	**γ 40 Gy**	**[illegible]10 immature**	**10~17**	**2~2.5**	**2**	**5**	**27~31**	**4~5, 6~8**	**Yellow**	**4**
**GP1***	**C-ion 10 Gy**	**[illegible]10 immature**	**4~6**	**1～2**	**1**	**5**	**26~30**	**4~5, 6~7**	**Yellowish green**	**5**
**Wild-type female**	**n/a**	**0**	**0**	**0**	**0**	**5**	**28~32**	**4~6.5, 7.5~9**	**Green**	**5**

†Pollen fertile in self-fertilization tests

* Incomplete hermaphrodites
