# Supplementary material for: Narrowing Down the Mapping of Plant Sex-Determination Regions Using New Y-Chromosome-Specific Markers and Heavy-Ion Beam Irradiation-Induced Y-Deletion Mutants in Silene latifolia
Source: G3 (Bethesda). 2012 Feb 1;2(2):271–8. doi: 10.1534/g3.111.001420 (PMC3284334; doi:10.1534/g3.111.001420)
Supplement: Supporting Information [file supp_2.2.271_TableS2.pdf]

Table S2 List of asexual mutants in this study

Genotype	Source	Stamen development†	Stamen development score
**ESS1**	**C-ion 100Gy**	**Suppressed at stage6**	**1**
**ESS3**	**C-ion 40Gy**	**Suppressed at stage5-6**	**0**
**ISS1**	**C-ion 100Gy**	**Suppressed at stage7-8**	**2**
**ISS2**	**C-ion 100Gy**	**Suppressed at stage7-8**	**2**
**ISS3**	**C-ion 100Gy**	**Suppressed at stage8**	**3**
**ISS4**	**γ 80Gy**	**Suppressed at stage7-8**	**2**
**ISS5**	**γ 80Gy**	**Suppressed at stage7-8**	**2**
**LSS1**	**γ 40Gy**	**Suppressed at stage 10-11**	**4**
**Wild-type male**	**n/a**	**No suppression**	**5**

†Stamen suppression stages are according to Grant *et al.* 1994
